# Pre-ICU length of hospital stay is a predictor of hospital but not ICU mortality

**DOI:** 10.1186/cc14607

**Published:** 2015-03-16

**Authors:** K Flavin, D Hall, G Marshall, P Zolfaghari

**Affiliations:** 1 Royal London Hospital, London, UK

## Introduction

Prolonged hospital stay prior to admission to the ICU was previously shown to be independently associated with poorer outcome [1,2]. This is probably due to slow deterioration of physiological function in hospital and influenced by processes leading to critical care admission [2]. We investigated whether commonly measured severity scoring systems (Acute Physiology and Chronic Health Evaluation (APACHE) II, Intensive Care National Audit and Research Centre (ICNARC) and Sequential Organ Failure Assessment (SOFA) scores) are significantly different in patients admitted with prolonged pre-ICU hospital length of stay, and describe mortality and hospital length of stay.

## Methods

A retrospective analysis of prospectively collected data of all emergency admissions in the ICNARC database of a 44-bed adult critical care unit within a major trauma centre over a 2-year period. Demographic data, APACHE II score, SOFA score, ICNARC model score, mortality, and length of hospital stay prior to and after ICU admission were collected. Five groups of patients were defined as follows: those admitted to ICU within 1 week of hospitalization (group 1), within 8 to 14 days (group 2), within 15 to 21 days (group 3), within 22 to 28 days (group 4), and more than 28 days (group 5). Chi-squared and ANOVA tests were performed using the SOFA statistics package.

## Results

A total of 2,248 emergency admissions were analysed. The majority of patients were admitted within 1 week of hospital admission (*n *= 1,897). They were younger and had lower APACHE II scores (15 vs. 19; *P *< 0.001). APACHE II scores were the same in all other groups (groups 2 to 5). Patients admitted to the ICU 3 weeks following hospital admission had significantly higher hospital mortality (up to 50%; *P *< 0.001) and ICU length of stay (12 ± 15 vs. 8 ± 10 days; *P *< 0.001). ICU mortality remained the same in all groups (20 to 28%). ICNARC and SOFA scores were equal between the groups. The post-ICU lengths of stay were significantly longer in groups 3 to 5. In-hospital CPR prior to admission to ICU was lower in patients from groups 4 and 5, probably signifying appropriate DNAR decisions made on the ward.

## Conclusion

Prolonged pre-ICU hospital admission is associated with higher hospital but not ICU mortality. Commonly measured scores do not reflect this higher mortality in patients admitted after prolonged stay in hospital. Further research into parameters that may reflect changes in physiological reserves may strengthen these scores for such patients.
